# The evolution of life size Barbie and Ken: Are they any closer to reality?

**DOI:** 10.1371/journal.pone.0351794

**Published:** 2026-06-16

**Authors:** Sara Grafenauer, Belinda Durey, Kevin Norton

**Affiliations:** 1 School of Health Sciences, Faculty of Medicine, University of New South Wales, Sydney, New South Wales, Australia; 2 Alliance for Research in Exercise, Nutrition and Activity (ARENA), Adelaide University, South Australia, Australia; Takushoku University, JAPAN

## Abstract

Barbie and Ken dolls have been criticized over time for their overt misalignment of body shape in comparison with the average adult, and the possibility that this impacts the formative minds of children, particularly girls. Updating a previous examination of the original Barbie and Ken dolls from 1996, this study compared the body measures of the 2016 “Fashionista” range—including Curvy, Petite, Tall, and Standard Barbie models, and new Malibu Ken—with representative population data of young adult females and males using anthropometric techniques. A range of anthropometric girth measures plus height were taken from each doll, and body proportions were calculated. The rules of allometry were used to scale the dolls to a standardized adult height of 170.18 cm to determine the dimensions the dolls at this adjusted size. The dimensions were then compared to the same height-adjusted reference population norms used in the original study. The comparison revealed significant alterations in the dolls’ morphology compared with the original Barbie and Ken, with key measures of waist-to-hip and chest-to-waist ratios becoming more realistic and falling within the 95% confidence range of the population. According to z-score deviation data, Curvy Barbie fell closest to the mean for most of the scaled girth measures, while new Ken was closer to the mean for all girths except ankle. Design changes reflect a shift toward greater body diversity, with potential benefits for children’s health behaviours through the production of a broader range of more diverse, human-like dolls. Importantly, key measurements and body proportions were generally closer to the respective reference populations.

## Introduction

Ruth Handler created Barbie at a time when most dolls were baby-like, with her aim of inspiring children, particularly girls, with dreams of adulthood. When the dolls were launched in 1959, Barbie had just two hairstyles and 36 different outfits. More recently, Mattel pre-empted a revived interest in Barbie, with a range of ‘Fashionista’ Barbie dolls and ‘new look’ Ken dolls. Mattel had long grappled with the body image issues associated with the doll, and Barbie is available now in different body types, skin tones, eye colours, hairstyles and from a launch range of seven, there are now 100’s of dolls [1]. Further mass-population interest has been inspired by the 2023 Barbie movie starring the Australian actress, Margot Robbie.

Norton et al (1996) and others have previously questioned the appropriateness of the body proportions of Ken and Barbie dolls, conducting quantitative analyses of typical anthropometric sites [2,3]. Although it has been acknowledged that Barbie was never modelled on the proportions of a real person, a number of studies have questioned the impact of the dolls perfectionistic appearance on children and adolescent body satisfaction / dissatisfaction, weight stigma, self-esteem and gender socialisation [4–9]. Research has also been extended to adult females, recalling accounts of Barbie-play from childhood and the impact this may have had on them now as adults [10]. Just two studies have researched the impact of the Fashionista Barbie, with the new range of body shapes and sizes [5,6].

Several studies of Barbie point to a need for society to address the potential body proportion and image concerns. Nesbitt and colleagues (2019) utilised digital images, rather than the actual dolls, shown to participants in minimal clothing [6]. The research focused on understanding the cognitive mechanisms that occur when girls are exposed to physique salient toys, in particular the self-matching based on social comparison theory. This theory asserts that in relation to our bodies, we compare ourselves to others (or via images) which can lead to negative evaluations and body dissatisfaction. In assessing the reactions from 6–9 year-olds and 10–14 year-olds the study utilised a self-reported questionnaire eliciting preferences for the particular images of the dolls, and body-part compatibility. The authors found preferences for some dolls over others and there were compatibility effects for all but the Tall Barbie from the Fashionista range and stated that ‘they [the dolls] remain unrepresentative of the diversity of female body shapes’ [6]. Harriger and colleagues (2019) provided physical versions of the Curvy, Tall and Petite dolls in identical bikinis, but replaced the heads, so all dolls appeared identical in terms of their facial features. Rather than using the new Standard Barbie from the Fashionista range, their research used an original Barbie. Participants aged 3–10 years assigned positive (happy, smart, has friends, pretty, helps others) and negative attributes (sad, not smart, has no friends, not pretty, mean) to each of the dolls with a preference for thin bodies and an aversion for larger bodies. The authors commend Mattel on the range, but at the same time express that the change may be insufficient to ‘combat pervasive messages about body size’ noting that both the Tall and Petite dolls remain extremely thin, and estimated that Curvy Barbie was still thinner than average [5]. Neither of these studies made any measurements of the dolls, and instead relied on visual cues for their assessment.

For consumers, both parents and children, new issues arise with new types of Barbie, particularly regarding the limited interchangeability of the available clothing, which was a consideration during the development and discussed by Mattel in the documentary depicting their decision-making and plan for the launch of the new range [1]. According to *Time Magazine*, Mattel declined to discuss the specific proportions of the new dolls or the process behind their redesign [11]. The new Ken and Barbie dolls also inspire a broader range of careers with Klamer (2023) assessing 92 Barbie dolls with medical and science careers, a tongue-in-cheek study of their accuracy in compliance with clinical and laboratory safety standards. They claimed that if Barbie were a real woman, she would hold numerous degrees, including many with PhD and MD-level qualifications, and boast a commendable resume, spanning many male-dominated fields [12], pointing to the aspirational nature of the doll. The range of new dolls reflects a further increase in diversity with representations of a variety of medical and health conditions and ethnic backgrounds.

Health professionals are increasingly concerned about body weight and weight stigma, with good reason as there has been a staggering increase in the global level of obesity since 1990 [13] alongside increases in disordered eating. To date, no studies have used a scientifically-validated process to assess the size and shape of the Fashionista range, although approximations of the life-size measurements of the dolls have been published in the lay media [14] together with assumptions that the body shapes remain unrepresentative. The current analysis provides an objective anthropometric assessment of the Fashionista range of dolls using methods described previously by Norton (1996). The aim was to compare the range of ‘Fashionista’ Barbies (Tall, Curvy, Petite and Standard), and new Ken with representative population data of young adult females and males using anthropometric techniques, and reveal the change in proportions in comparison to the original dolls.

## Materials and methods

The anthropometric landmarks were the same as those used in the original study [3] and were identified by two independent anthropometrists on all dolls used. In addition to height, the following girth measurements were made according to international guidelines [15]: neck, chest, waist, hip, thigh, calf, ankle, upper arm, forearm, and wrist. Body proportions for waist:hip (WHR), chest:waist (CWR), and chest:hip (CHR) were calculated as in the original study. Furthermore, waist:height (WHtR) ratios were also calculated so that doll proportions could be compared to background population trends calculated from NHANES USA datasets 1961–2018 [16].

In this study, four current Barbie dolls and one Ken doll of the Fashionista range were used. An original Barbie was used to check agreement with previous measures, and because anthropometry measurements vary slightly due to small differences in the size of individual dolls. Duplicate measures were taken on all dolls at all sites by two separate anthropometrists, and averages were used in all subsequent analyses.

The same reference populations were used for comparisons in the original [3] and current studies for WHR and CWR. This allowed trends in doll morphology to be analysed against a stable reference group and independently of background population changes. The original reference groups were based on values from 135 young females and 50 males (18–35 years) who represented a relatively active cross section selected from a population of South Australian university students (SAS) [3]. Both reference groups were of predominantly Anglo-Australian ethnicity.

To determine differences between all dolls and evolving population trends, the NHANES datasets were used to calculate WHtR at regular intervals from 1961–2018 for both males and females (20–29 years). This was possible because these anthropometry sites are routinely measured in large USA population surveys [16].

All individual anthropometry measurements were scaled using classical allometry (where lengths, girths and breadths are proportional to height) to a common height of 170.18 cm so that direct comparisons between the groups could be made. The height is a standard international “Phantom” height for anthropometry scaling studies [15]. Barbie doll values were compared with the female reference groups, while Ken doll values were compared with the male reference groups.

All anthropometry values were expressed as z-score deviations where a zero score indicated the doll measurement was identical to the reference population. Measurement reliability was determined by repeat measures on two dolls by each of the anthropometrists. The reliability of the intra- and inter-tester measurements was reported as the technical error of measurement (TEM) [15].

## Results

Intra-tester technical errors of measurement (TEMs) were 1.4% and 1.8% for girth measures, and 0.5% and 0.6% for length measures, for the two anthropometrists, respectively. Inter-tester TEMs were 2.6% and 2.3% for girths and lengths, respectively.

**Fig 1:**
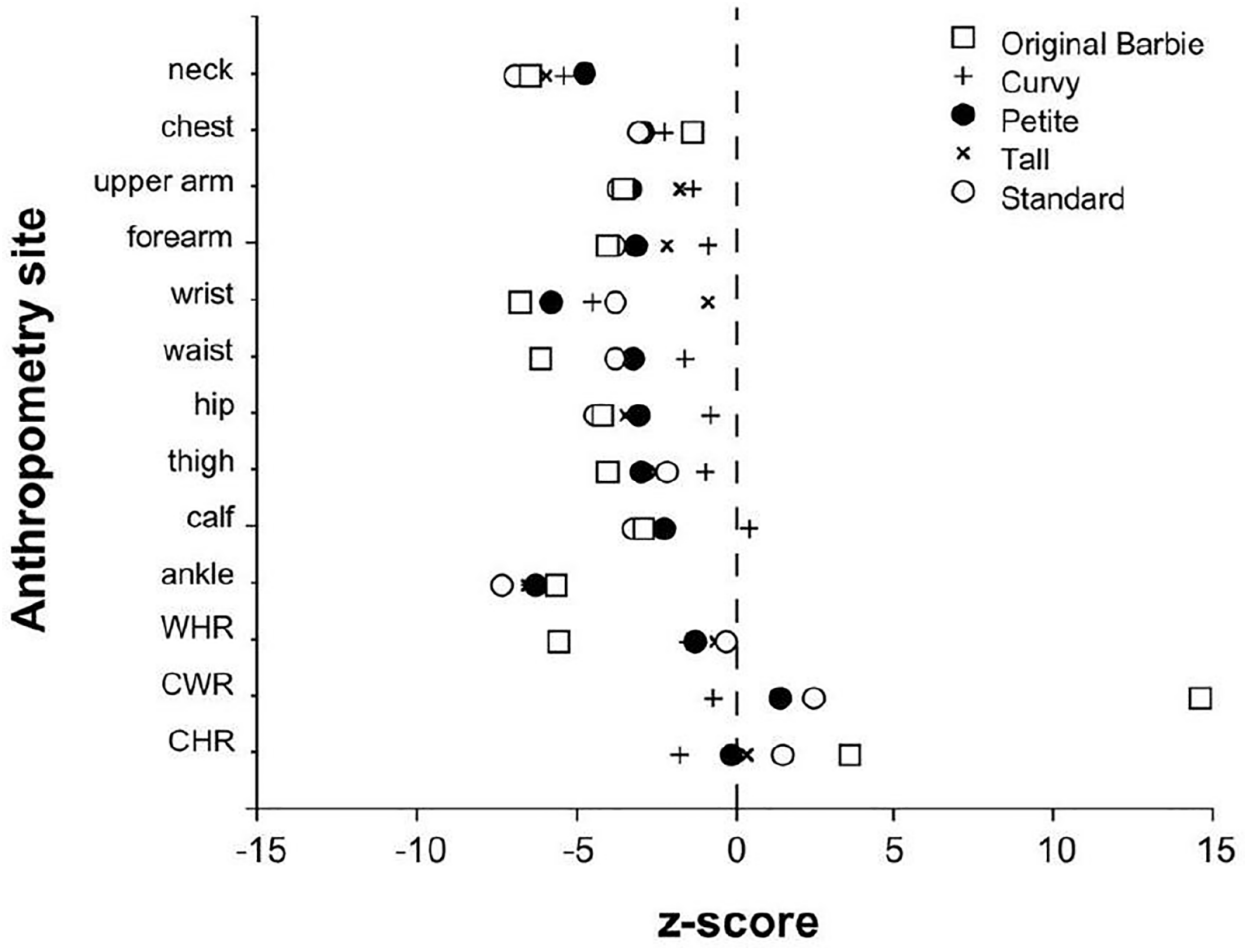
The girth and ratio measurement deviations (z-scores) for the original and Fashionista Barbie dolls relative to the SAS reference female population. WHR = waist: hip ratio, CWR = chest: waist ratio, CHR = chest: hip ratio.

**Fig 2:**
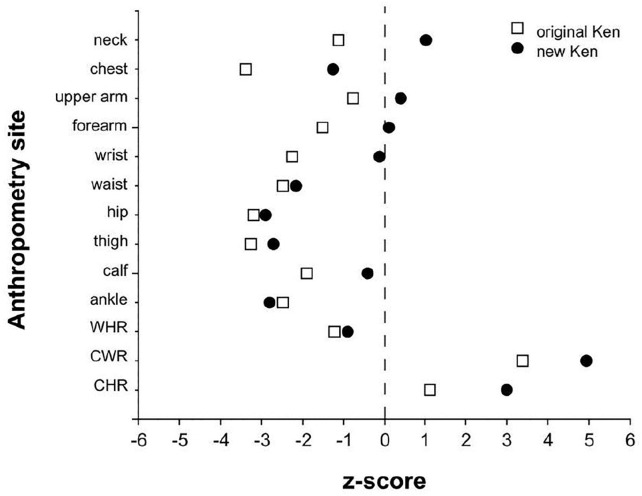
The girth and ratio measurement deviations (z-scores) for the original and Fashionista Ken dolls relative to the SAS reference male population. WHR = waist: hip ratio, CWR = chest: waist ratio, CHR = chest: hip ratio.

Tables 1 and 2 present the absolute values (in cm) for all anthropometric variables across all groups, once scaled to the common height of 170.18 cm. Figures 1 and 2 display the z-scores for girth and ratio measurements for the Barbie and Ken dolls, respectively. These are shown relative to the original South Australian student (SAS) reference populations [3].

**Table 1:** Anthropometric measurements of all Barbie dolls used in this study and the SAS reference group when scaled to a common height of 170.18 cm (data are reported as mean ± standard deviation, in cm except the ratios). The very small standard deviations reported for doll measurements reflect repeated measurement precision.

	Original Barbie Mean SD		Curvy Mean SD		Petite Mean SD		Tall Mean SD		Standard Mean SD		Reference Mean SD	
**Neck**	23.9	0.4	25.1	0.2	25.8	0.4	24.4	0.1	23.1	0.2	32.7	1.4
**Chest (bust)**	82.3	1.2	78.1	0.2	74.4	0.6	74.6	0.1	73.6	0.1	90.3	5.5
**Upper arm**	20.2	0.3	24.9	0.4	20.9	0.1	24.1	0.1	20.0	0.2	27.8	2.1
**Forearm**	18.7	0.3	23.6	0.5	19.9	0.3	21.4	0.1	18.8	0.2	24.9	1.6
**Wrist**	10.6	0.3	12.5	0.4	11.5	0.1	15.4	0.1	13.1	0.2	16.1	0.8
**Waist**	40.7	0.6	62.2	0.3	54.7	0.5	55.0	0.1	52.0	0.1	69.8	4.7
**Hips**	72.7	0.6	93.7	0.1	81.5	0.5	79.4	0.8	74.0	0.1	97.9	5.4
**Thigh**	42.6	0.9	54.1	0.5	46.3	0.2	48.1	0.0	49.3	0.2	57.5	3.8
**Calf**	29.3	0.6	37.1	0.0	31.0	0.1	30.4	0.8	28.8	0.2	36.1	2.3
**Ankle**	15.6	0.3	14.3	0.4	14.5	0.1	14.2	0.3	13.3	0.0	22	1.2
**WHR**	0.56	0.01	0.66	0.00	0.67	0.01	0.69	0.01	0.70	0.00	0.71	0.03
**CWR**	2.02	0.01	1.26	0.01	1.36	0.00	1.36	0.00	1.41	0.01	1.29	0.05
**CHR**	1.1	0.01	0.83	0.00	0.91	0.01	0.94	0.01	0.99	0.00	0.92	0.05

**Table 2:** Anthropometric measurements of the Ken dolls used in this study and the SAS reference group when scaled to a common height of 170.18 cm (data are reported as mean ± standard deviation, in cm except the ratios). The very small standard deviations reported for doll measurements reflect repeated measurement precision.

	Original Ken Mean SD		Malibu Ken Mean SD		Reference Mean SD	
**Neck**	32.1	1	36.1	0.1	34.2	1.9
**Chest**	75	3.3	85.2	1.4	91.2	4.8
**Upper arm**	27.1	0.9	29.7	0.5	28.8	2.2
**Forearm**	23.6	1	26.2	0.1	26	1.6
**Wrist**	14.6	0.5	16.3	0.4	16.4	0.8
**Waist**	56.5	1.9	59.9	0.1	80.9	9.8
**Hips**	72	2.6	74.0	0.2	93.7	6.8
**Thigh**	41.3	1.4	43.3	0.1	53	3.6
**Calf**	31.6	1	34.6	0.5	35.4	2
**Ankle**	18.3	0.7	17.9	0.2	21.5	1.3
**WHR**	0.78	0.01	0.81	0.00	0.86	0.06
**CWR**	1.33	0.02	1.42	0.02	1.13	0.06
**CHR**	1.04	0.01	1.15	0.02	0.97	0.06

Fig 1 demonstrates that the Barbie Fashionista dolls fall closer to the SAS population mean compared to the original doll for most measures, including key body proportions. Among them, the Curvy Barbie most closely reflects the SAS population distribution. Similarly, the new Ken doll exhibited z-scores that were closer to the population mean for all body sites except the ankle, although only one of the three body proportions aligned closely, as illustrated in Fig 2.

**Fig 3:**
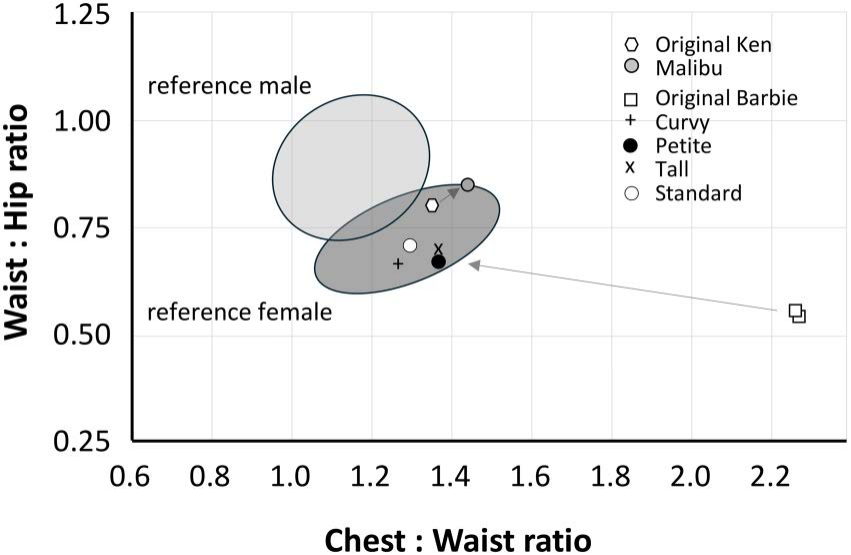
The relationship between the waist: hip ratio versus the chest: waist ratio for the Barbie and Ken dolls. Representative population distributions are shown as grey ellipses for both young adult males and females.

**Fig 4:**
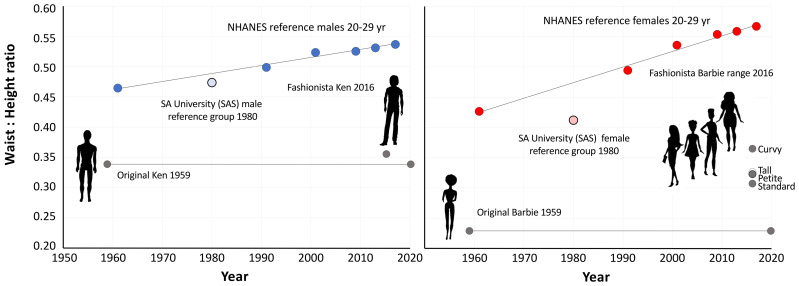
The figure shows the WHtR for both Ken and Barbie dolls over time, relative to background reference population measures. The reference populations are 20-29 year old males and females from the large NHANES datasets within the USA 1961–2018 [16].

Fig 3 shows the relationship between the waist: hip ratio versus the chest: waist ratio for all dolls, each doll type shown as a unique symbol. The two larger grey population distribution ellipses are superimposed and illustrate where the 95% confidence intervals are located for the background SAS reference populations of young adults females and males, respectively [3]. Fig 4 illustrates the body shapes of the background NHANES-sampled young adult USA population has changed considerably over time in line with overweight and obesity trends [13]. The figure illustrates the unrealistically low ratios for the original Ken and Barbie dolls from 1959, which would be approximately <1 in 10,000,000 (z = −5.19). The new Fashionista range introduced in 2016 all have a greater WHtR compared to the original dolls, but are still relatively extreme (low) compared to population norms, approximately 1 in 1000 (z = −3.2).

The doll values are plotted relative to the respective background SAS reference populations shown as ellipses. The ellipses represent the 95% CI for the reference populations. Note there are two measures shown for each original Barbie and Ken doll. These are the measures taken during the first and updated studies, respectively. Contemporary population reference norms that included chest, hip and waist girth measures were not found.

## Discussion

This study used standard anthropometric procedures to measure body size and proportions of updated Barbie and Ken dolls from the Fashionista range. The measures were compared to those taken on original doll body types, as well as against background, young adult reference populations, after scaling all groups to a common height. It was clear from this study that very significant alterations in the Fashionista Barbie dolls’ morphology have occurred since measurements were taken of the original Barbie in 1996 [3]. As shown in Table 1 and Fig. 2, Barbie’s dimensions across 80% of the anthropometric sites, and 100% of the proportions measured, have shifted closer to the reference population. Of note, the controversial focus on Barbie’s bust girth was, and continues to be unfounded, with negative z-scores as shown in Fig. 2.

The key proportions of waist: hip and chest: waist have evolved to be more realistic such that all four new Barbie dolls now fall within the 95% confidence ellipse for the young adult reference group (Fig 3). For Ken’s scaled dimensions, 90% of the measures were closer to the reference population (Table 2), while only one of the three proportions moved closer to young adult males (Fig 3). Ken’s proportions still remain outside the 95% confidence range of the reference population, almost entirely due to his persistently narrow waist as reinforced in Fig 4.

The positive changes in Barbie and Ken, including for many of the individual body sites, should serve a degree of reassurance for health professionals and parents making purchases of these dolls. Although they are “just dolls”, Mattel promotes them as ‘aspirational role models’ and their body shapes and proportions (especially their proportions) are meaningful to people who play with them [10]. Since its introduction in 1959, the Barbie brand has sold over one billion units and has remained one of Mattel’s largest revenue contributors, however the evolution of the doll in an increasingly digital world and play-space was critical. After a documented decline in sales during the mid‑2010s, Barbie revenues recovered from 2018 onward, a period that coincided with the introduction of dolls featuring more diverse body shapes and identities [17]. Ynon Kriez, Chairman and CEO, Mattel Inc. was the driving force behind the 2023 Barbie initiatives including the Barbie Movie. “Under his leadership, *Barbie* became a $1.4 billion global success, validating his strategy of expanding Mattel’s brands into film, television, and digital gaming” [18] and remains in the top three of toy brands [19] with sales close to, or in excess of 1 billion US dollars annually [20]. Ken dolls are marketed within the Barbie brand and do not have separately reported longitudinal sales figures. More recently Mattel has chosen to utilise themes important in current society, regarding diversity in appearance and in reflecting a range of medical issues, including most recently a Type 1 Diabetes Barbie (complete with insulin pump and continuous blood glucose monitoring), the range in body shapes, more reflective of the population, is important in promoting healthy views among children [4–7].

Interestingly, Harriger et al. (2019) titled their paper ‘You can buy a child a Curvy Barbie doll, but you can’t make her like it’, indicating the power of the consumer within this competitive environment. Although most of Curvy Barbie’s measurements are now closer to the population mean (that is, z-scores closer to zero), girls in this study identified Curvy Barbie as the doll they least wanted to play with, and there remained a preference for the Barbie types with a thin body [5]. The children participating in the study referred to the Curvy Barbie as “big”, “fat” or “chubby”, favouring instead the Tall Barbie who they felt was more “grown up”, perhaps a reflection on their own height in relation to adult height measurements. Before the launch of the Fashionista range, Worobey and Worobey (2014) had created Barbie-like dolls that had been altered to represent thin, average and ‘fat’, and found the same tendency to attribute positive characteristics to the thin and average doll, and negative attributes to the ‘fat’ doll [9]. Using Fashionista dolls, Nesbitt et al. (2019) found that participants thought Curvy Barbie was the most ‘likable’, yet the least desirable and least attractive, while Original and Tall Barbie were thought to be less pleasant, yet more highly desirable and attractive. Petite Barbie was the most preferred overall, which the authors suggest may be due to greater ‘self-relevance’ [6], that is, smallest height, and therefore more relatable to the study participants. This is supported by Table 1, which shows that, after scaling, Petite Barbie is simply a smaller version of Standard and Tall Barbie. Our analysis points to opportunities for further revisions to the dolls and this should be a consideration in order to not expose children to unnecessary scrutiny of their bodies as they grow and develop.

In an effort to help consumers understand the new Fashionista range, Bates (2016) presented an article in *BBC News Magazine* with an extrapolation for each doll indicating the most aligned clothing size, suggesting that Curvy Barbie would be a UK size 6/8 waist and 8 hips (US size 4/6); Tall Barbie would be a UK size 4 (US size 2); Petite Barbie a size 2 waist and a size 0 hips (US size 0/2) [14]. Although these data have been widely referenced, they are based on an unscientific crude approach based on multiplication of body measurements by estimated 6:1 ratio to give a ballpark extrapolation to adult height. In contrast, our method used standardized scaling methods to compare measured dimensions at the same body height (170.18 cm), rather than by approximation. It is through this objective scientific method that most of the Fashionista Barbie and new Ken proportions, and their individual body measurements, are shown to be more realistic relative to the previous iterations of the dolls.

Harriger et al. (2019) acknowledged that Barbie ownership was not related to body dissatisfaction among younger or older children in their study, nor was it specifically related to the number of positive or negative adjectives assigned. Similarly, an Australian study using the original Barbie in print form, compared with physical engagement and physical observation, found that while interaction with Barbie promoted a preference for a thin ideal, the doll did not impact self-esteem or body image [14]. The concern expressed in the literature is that a thin ideal, set at an early age, is likely to remain stable and serve as a precursor to later body dissatisfaction [4–7]. However, there are many sociocultural sources, beyond interactions with Barbie, that reinforce perceptions of body weight and shape, and they are everywhere: in television and magazine advertising, depicted in movies, shop mannequins displaying fashion items, and now on social media, but also through peer and family interactions [4].

More recent launches within the Barbie range now allow the Barbie characters a greater range of motion with additional joint movement. Designed with 22 “joints,” the latest Barbie ‘Made to Move’ dolls can bend at the neck, upper arms, elbows, wrists, torso, hips, upper legs, knees, and ankles. Interestingly, the physicality of the dolls is thought to be a positive aspect that may be protective against appearance-centric issues discussed in a range of studies [7]. This is particularly noted when studies have used actual dolls rather than digital images [6]. As suggested by Rice et al. (2020), promoting play with Barbie that focused on participant perceptions of functionality may provide a protective effect against body dissatisfaction in children. It may be what the doll can do, rather than how they look that matters. Through research involving adult women and the way they remember Barbie, and the aspects that are recalled from childhood in the context of ‘play’, also adds to the body of research in this area [10]. Rather than impacting body image or dissatisfaction, the women recalled positive experiences of designing and making clothes, building houses, and planning out elaborate scenarios where Barbie played a key role in aspirational careers as suggested by Klamer [12], rather than just playing a mother with a baby-like doll which prior to Barbie in 1959, was the only option.

One potential limitation of this study is that the original SAS reference group participants, having volunteered for body composition studies, were therefore likely to be leaner than randomly selected young adults. For example, waist girth in young adult females and males (18–24 yr) in the 1995 Australian National Survey averaged 73.4 and 84.4 cm, respectively, versus the university student volunteers who had waist girths of 69.8 and 80.9 cm, respectively. It is also important to note that background population values for virtually all body dimensions and proportions have changed considerably in the time between studies; perhaps best illustrated by the Australian 2022 waist girth measures which had increased to 81.3 and 88.8 cm, respectively [21,22]. Similar dramatic rates of change in population proportions are illustrated by the USA population trends in Fig 4.

## Conclusions

Substantial alterations in the Fashionista dolls’ morphology have been made since measurements were taken of the original dolls in 1996. In general, for the Barbie range, individual girth measures and the three described proportions have become more realistic and aligned closer with human-like dimensions. For the Ken doll, individual girth measures have also approached the reference population, while proportion measures have moved slightly further away. This study indicates the importance of measurement rather than assumptions, and the use of corrected anthropometric approaches to the required measurement. For parents and health professionals, there is more to be done to promote a healthy body weight and prevent stigmatised views of body shape as children grow and develop. Despite the improvements reported here, there is a need for continued scrutiny and dialogue around the impact of toy design on body image, and this may extend beyond the scope of this study, for example make-up and clothing design. Future iterations should consider, and perhaps also test, the impact on psychological and developmental issues, helping to reinforce the positive health messages protecting young audiences.

## Supporting information

S1 File Barbie II study. (XLSX)
